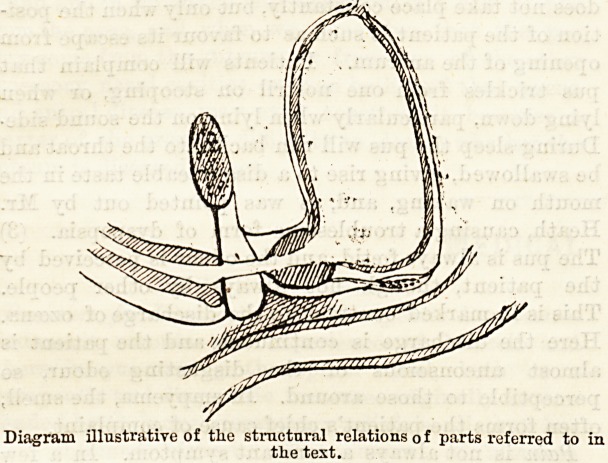# On the Treatment of Rupture of Urethra

**Published:** 1894-03-10

**Authors:** Alex. Miles

**Affiliations:** Lecturer in Anatomy, Queen's College, Belfast


### Medical Progress and Hospital Clinics.

[*The Editor will be glad to receive offers of co-operation and contributions from members of the profession. All letters should be addressed to* The Editor, The Lodge, Porchester Square, London, W.]

### ON THE TREATMENT OF RUPTURE OF URETHRA.

Alex. Miles, M.B.C.M.Edin., Lecturer in Anatomy, Queen's College, Belfast.

The urethral canal may be ruptured from various causes and in different situations, and the treatment of the resulting extravasation of urine varies according to circumstances. Success in the treatment depends upon an accurate knowledge of the anatomical relation of the parts implicated, which alone renders a correct diagnosis possible.

Amongst the more common causes of rupture of the urethra may be mentioned (1) stricture of the canal, the presence of which dilates and weakens the part behind, and so renders it liable to give way when there is any excess of pressure. When due to this cause, oftener than not the urine is already contaminated by septic organisms before the accident happens, and the escape of this infective fluid into the tissues of the perineum

Diagram illustrative of the structural relations of parts referred to in the text.

necessitates prompt measures of treatment being adopted, and warrants a grave prognosis being given. Even when the bladder contents are aseptic, although the chances of recovery are greater, no delay in treatment is permissible; (2) falling on a sharp instrument (such as broken glass or china), causing a clean cut of the urethra, is a not uncommon accident to children If treatment by passage of an instrument be adopted at once there is little risk of the symptoms becoming alarming. The urethral wound heals, no urine escapes, and the frequent passage of a bougie subsequently obviates the formation of a stricture; (3) a fall on the fork, astride a bar or rope, often leads to tearing of the urethra, without any damage to the skin; and (4) a similar condition of affairs results after some fractures of the pelvis where spicules of bone perforate the tube. In these cases the symptoms are more severe, bleeding from the orifice, with escape of urine into the tissues of the perineum, being among the most prominent, and the appropriate treatment depends on collateral circumstances.

The course which the urine follows when it escapes from the canal is regulated by the fascia of the perineum, which it is now our business to consider. The upper part of the arch of the pubis is filled in by a dense fibrous membrane — the triangular ligament. Behind this is another fibrous layer, a portion of the pelvic fascia, and in front of it is a layer of fascia—Colles' fascia, which is derived from the superficial fascia. At the base of the triangular ligament these three layers blend. The different parts of the urethra bear a definite relation to each of these fasciæ. The prostatic portion lies behind the pelvic fascia; the membranous portion between the pelvic fascia and the triangular ligament; and the spongy portion in front of the triangular ligament, between it and Colles' fascia.

When the lesion occurs in the prostatic urethra, one of two courses is open to the urine—it may either pass backward and point in the region of the anus, whence a free incision will remove it, or it may pass upward, through the layers of the pubo-prostatic ligament, and spread in the loose subperitoneal tissue between the fascia transversalis and the bladder wall. In this situation the prognosis is grave, and the treatment consists in the passage of a soft catheter into the bladder and its retention there; early and free incision above the pubis in the middle line, and the adoption of every means to keep up the strength of the patient by food, stimulants, and internal antiseptics.

Urine extravasated through the membranous urethra occupies a pouch, bounded in front by the triangular ligament, behind by the pelvic fascia, below by the blending of these, above by the body of the ospubis, and laterally by the pubic arch. So long as these boundaries remain intact, the only symptoms are intense localised pain due to the tension and a circumscribed bulging in the perineum. The treatment is obviously to pass a catheter, and allow the effused fluid to escape by a perineal incision. When the posterior boundary gives way, the condition becomes equivalent to rupture in the prostatic portion, and when the triangular ligament yields to rupture of the spongy urethra.

These situations for extravasation are by no means so common as that which remains, namely, in front of the triangular ligament, and here the fascial attachments are more complicated. The deep layer of the superficial fascia—fascia of Colles—we have already seen to blend with the base of the triangular ligament, and so to shut off the posterior part of the perineum from the anterior part. It is also attached along the rami of the pubis and ischium on each side, thus preventing the passage of urine down the thighs. The only open course is into the scrotum, and thence along the spermatic cords on to the anterior abdominal wall.

In all cases the first step in treatment is to instruct the patient to abstain from voluntary attempts at micturition, until a soft catheter has been passed into the bladder. Should all attempts at this fail, as sometimes occurs, then the effused urine must be evacuated by freely incising the tissues in which it is contained, and subsequently using antiseptic (carbolic) fomentations. Further extravasation is prevented by performing perineal section. The diminution of the swelling on account of the incisions will usually permit of the use of Syme's staff, and the urethra will be readily reached and opened and the bladder drained. Sometimes no instrument can be passed beyond the seat of rupture, and Wheelhouse's operation of cutting down and finding the proximal end will be necessary —an operation of great difficulty and requiring much skill and time.

When the ruptured urethra is exposed, an attempt should be made to unite the ends with a view to obtaining rapid union and diminishing the risks of subsequent stricture. In all cases of extravasation of urine, great care is necessary to keep up the strength of the patient by appropriate food and stimulants; to keep the skin, bowels, and kidneys working well, and to prevent or counteract sepsis by internal as well as local antiseptics.

## Figures and Tables

**Figure f1:**